# Bioavailability and biodistribution of differently charged polystyrene nanoparticles upon oral exposure in rats

**DOI:** 10.1007/s11051-015-3029-y

**Published:** 2015-05-22

**Authors:** Agata P. Walczak, Peter J. M. Hendriksen, Ruud A. Woutersen, Meike van der Zande, Anna K. Undas, Richard Helsdingen, Hans H. J. van den Berg, Ivonne M. C. M. Rietjens, Hans Bouwmeester

**Affiliations:** Division of Toxicology, Wageningen University, Tuinlaan 5, 6703 HE Wageningen, The Netherlands; RIKILT Wageningen UR, P.O. Box 230, Akkermaalsbos 2, 6700 AE Wageningen, The Netherlands; TNO Earth, Life and Social Sciences, Princetonlaan 6, 3584 CB Utrecht, The Netherlands

**Keywords:** Polystyrene nanoparticles, Surface properties, Biodistribution, Bioavailability, In vivo, Oral exposure

## Abstract

The likelihood of oral exposure to nanoparticles (NPs) is increasing, and it is necessary to evaluate the oral bioavailability of NPs. In vitro approaches could help reducing animal studies, but validation against in vivo studies is essential. Previously, we assessed the translocation of 50 nm polystyrene NPs of different charges (neutral, positive and negative) using a Caco-2/HT29-MTX in vitro intestinal translocation model. The NPs translocated in a surface charge-dependent manner. The present study aimed to validate this in vitro intestinal model by an in vivo study. For this, rats were orally exposed to a single dose of these polystyrene NPs and the uptake in organs was determined. A negatively charged NP was taken up more than other NPs, with the highest amounts in kidney (37.4 µg/g tissue), heart (52.8 µg/g tissue), stomach wall (98.3 µg/g tissue) and small intestinal wall (94.4 µg/g tissue). This partly confirms our in vitro findings, where the same NPs translocated to the highest extent. The estimated bioavailability of different types of NPs ranged from 0.2 to 1.7 % in vivo, which was much lower than in vitro (1.6–12.3 %). Therefore, the integrated in vitro model cannot be used for a direct prediction of the bioavailability of orally administered NPs. However, the model can be used for prioritizing NPs before further in vivo testing for risk assessment.

## Introduction

The number and range of consumer products containing nanoparticles (NPs) is constantly growing, examples ranging from e.g. lipsticks, toothpaste, food additives, health supplements and juice clarifiers to food packaging materials (Chaudhry et al. 2008; PEN 2013, Bouwmeester et al. 2014). Therefore, the likelihood of oral exposure to NPs is still increasing. Thus, there is a need to develop methods to assess the oral bioavailability of NPs. For scientific, ethical and economical reasons, in vitro models are desired.

To assess the performance of in vitro models, NPs need to be selected that differ in translocation efficiency. In several studies, polystyrene NPs (PS-NPs) have been shown to translocate across in vitro intestinal barrier models (Walczak et al. 2014; Kulkarni and Feng 2013; Martinez-Argudo et al. 2007), and this translocation depended on NP characteristics like size, charge and surface chemistry. In addition, in vivo studies have demonstrated the bioavailability of PS-NPs after oral exposure as well (Jani et al. 1989, 1990; Hussain et al. 1997). However, no data are available from studies that used the same PS-NPs in vitro and in vivo. With the present in vivo study, we aim to evaluate the validity of our in vitro model by comparing the newly obtained data with the previously obtained in vitro data. To all this comparison, we used the same PS-NPs as used in our previous in vitro studies (Walczak et al. 2014).

In these previous in vitro studies, we developed an integrated in vitro gastrointestinal digestion and in vitro intestinal epithelium model as a screening tool for assessing the translocation efficiency of orally administered PS-NPs. The translocation of these PS-NPs ranged from 1.6 to 12.3 % (Walczak et al. 2015). Furthermore, these results indicated that the translocation rate of the PS-NPs was affected by at least three factors: (i) the physicochemical properties of the PS-NPs (i.e. size and surface chemistry) (Walczak et al. 2014), (ii) the environmental conditions that the PS-NPs were exposed to (i.e. incubation in an in vitro gastrointestinal digestion model) (Walczak et al. 2015) and (iii) the properties of the in vitro monolayer simulating the intestinal epithelium (i.e. presence of mucus) (Walczak et al. 2014). Effects of size, surface chemistry and the properties of the in vitro monolayer on PS-NP translocation have also been reported by others (Hussain et al. 2001; Szentkuti 1997; des Rieux et al. 2005; Fazlollahi et al. 2011; Mahler et al. 2012).

In order to reduce the undesirable use of animals in the evaluation of uptake of surface-modified NPs (Hartung et al. 2013), alternative in vitro intestinal translocation models need to be developed. However, before such in vitro models can be used in a risk assessment of NPs, they need to be validated (Kandarova and Letasiova 2011; Worth and Balls 2004) using in vivo data (Genschow et al. 2002). As mentioned earlier, the aim of this study was to assess to which extent our in vitro model (combination of a gastrointestinal digestion model and an intestinal epithelium translocation model) predicts the translocation efficiency occurring in vivo. To that end, rats were orally exposed to a single dose of the same PS-NPs as used in previous in vitro studies [i.e. neutral, positive and negative, the latter from two different suppliers with different surface modifications (Walczak et al. 2014)] and PS-NP bioavailability in organs was determined.

## Materials and methods

### Nanoparticles

“Neutral”, amine- and carboxyl-modified 50 nm PS-NPs [referred to as 50 (0), 50 (+), 50 (−M)] with a red fluorophore core (Ex/Em: 530/590) were purchased from Magsphere (Pasadena, CA, USA). Carboxyl-modified 50 nm PS-NPs (referred to as 50 (−P)) with a yellow-green fluorophore core (Ex/Em: 485/530) were purchased from Polysciences (Warrington, Pennsylvania, USA). PS-NPs were washed prior to administration by centrifugation for 5 h at 18,000×*g*, 15 °C and re-suspension in deionised water, in order to remove preservatives and surfactants present in the suspension solution. The final mass concentration of all stock suspensions was 2.5 %. Throughout this paper, we have chosen to use the manufacturer indication to identify the different types of NPs.

### NP characterization

The NPs, as purchased, were characterized previously (Walczak et al. 2014) using Scanning Electron Microscopy (SEM), Dynamic Light Scattering (DLS) and zeta potential measurements. To confirm the size of the PS-NPs as administered to animals (i.e. following washing and re-suspension in deionised water), hydrodynamic sizes were again determined using DLS. The measurements were performed as previously described (Walczak et al. 2012). Suspensions of 100 µg/ml were analysed in triplicate, and the results are presented as the mean ± SD.

### Stability of the fluorescent dye in PS-NPs during exposure

No detectable leakage of the fluorescent dye from the used PS-NPs was shown upon incubation under simulated gastric digestion conditions. For this, PS-NPs were incubated in simulated gastric juice for 2 h at 37 °C, after which the suspensions were brought to neutral pH and suspensions at 250 µg/ml were centrifuged (30 min, 3000×*g*, 20 °C) in filter tubes (Amicon Ultra-4 3 kDa Ultracel-PL memb 24/Pk; Millipore BV, Netherlands). The filtrates were then analysed for fluorescence.

### Animal experiment

Five-week-old male Fischer 344 rats with a body weight of 107 ± 8 g (upon arrival) were obtained from Harlan (Horst, The Netherlands). Upon arrival, rats were left to acclimatise for three weeks in groups of two under standard conditions of humidity (55–65 %), temperature (22 ± 3 °C) and light (12-h light/12-h dark cycles), with ad libitum access to feed pellets (Abdiets, Woerden, The Netherlands) and tap water. After 3 weeks, 25 rats were divided into five groups (*n* = 5) for the experiment, based on their weight to have a similar weight distribution in each group (201 ± 13 g). Before treatment, rats were fasted for 2 h. A single dose of 1 ml PS-NP suspension per 200 g bw was administered through oral gavage at a concentration of 25 mg/ml (resulting in a dose of 125 mg/kg bw). Rats in the control group received the same volume of vehicle solution (i.e. deionised water) only. The dose of 125 mg/kg bw was selected as this was the highest achievable dose with these NPs. We selected this rather high dose to increase the likelihood of generating detectable amounts of NPs in tissues given the expected low uptake in rats. After administration, rats were housed separately until the end of the experiment. All animal experiments were approved by the ethical committee on animal experimentation of Wageningen University & Research centre, The Netherlands.

Blood samples (around 100 µl) were withdrawn from the tail vein at time points: 0, 0.5, 1, 2 and 4 h and collected in heparinized tubes. At *t* = 6 h, rats were sacrificed under anaesthesia, and blood was collected from the aorta, after which liver, kidneys, spleen, lungs, heart, testis, brain, stomach, small intestine and large intestine were collected. Previous studies indicated that 6 h after an oral administration of NPs, their plasma levels were declined (Lee et al. 2012; Bhattacharjee et al. 2013). In addition, after 6 h the administered bolus has passed the small intestine (Fallingborg et al. 1989; Durmus-Altun et al. 2011). Food remainders and faecal contents were gently removed from the stomach and small- and large intestines with a spoon, and the tissues were subsequently rinsed in PBS, to remove any unabsorbed PS-NPs. The organs were weighed and divided into two pieces for fluorescent and histopathological evaluation. The pieces meant for fluorescence measurements were preserved on ice, and the pieces meant for histopathology/microscopic observations were preserved in Bouin solution (testis) or in 10 % neutral buffered formalin (all other organs).

### Fluorescence measurements of blood and organs

Harvested tissue samples (organs and blood) were digested using an aqueous enzyme solution containing 1 g/l proteinase K (Sigma-Aldrich, St. Louis, MP, USA) in 50 mM NH_4_HCO_3_ buffer (to maintain a constant pH value of 7.4 during enzymatic digestion) and 5 g/l SDS to improve activity of the enzyme (Loeschner et al. 2013). Organs were carefully weighed, cut into pieces and digested in digestion buffer at a weight ratio of 1:5. The samples were thoroughly vortexed and incubated at 37 °C under continuous stirring on a magnetic stirrer for 4 h. This resulted in slightly turbid but homogenous suspensions. Fluorescence of the samples was measured using a SpectraMax M2 microplate reader (Molecular Devices, Berkshire, UK) at excitation/emission wavelengths of 530/590 nm and 470/520 nm, for red and yellow-green PS-NPs, respectively. The PS-NP concentration was determined based on previously prepared standard calibration curves in each organ separately, obtained by spiking blank organ homogenates (prepared as described above) with serial dilutions of PS-NPs ranging from 0 to 20 µg/ml (for organs) and 0–80 µg/ml (for blood). Calibration curves were slightly non-linear at very low concentrations. For blood samples, calibration curves were linear from NP concentrations of 5 µg/ml (for 50 nm (0) and 50 nm (−M) NPs) and 1 µg/ml for the other NPs. For other tissue samples, calibration curves were linear from the NP concentrations of 1 µg/ml. Above this concentration, calibration curves were linear for all tissue samples, except for the kidney-derived samples, but amounts of NPs in organs could be derived in all cases. The PS-NP bioavailability was estimated by summing up the amounts of NPs measured in all tested organs except for the brain, stomach wall and small- and large intestinal walls. For this, the amounts of NPs per gram tissue were multiplied by the weights of the organs.

### Histopathology

Samples for histopathology, fixed in 10 % formalin or Bouin solution, were dehydrated in a series of ethanol and embedded in paraffin. Approximately 5-μm-thick sections were cut, mounted on glass slides and stained with hematoxylin and eosin (H&E). The sections were observed under an optical microscope (Zeiss, Cambridge, UK) at different magnifications.

### Fluorescence imaging

Intact livers, kidneys, spleens, lungs, testes, small intestinal wall and large intestinal wall were scanned for fluorescence with a fluorescence imager (Cellavista V3.1, SynenTec Bio Services GmbH, Münster, Germany) using illumination at wavelengths Ex/Em = 470/520 nm or 530/590 nm, for yellow-green (−P) PS-NPs and red (0, +, −M) PS-NPs, respectively.

### Statistics

Data were analysed with SPSS (IBM, Version 21), and the charts were generated with Prism software (v5.02; GraphPad Software, Inc., La Jolla, USA). A one-way analysis of variance ANOVA test and post hoc Tukey test were used to determine significant differences between the groups.

## Results

### NP characterization

The PS-NPs with different surface modifications were characterized in water using SEM and zeta potential measurements as reported previously (Walczak et al. 2014). Briefly, all types of 50 nm PS-NPs had similar size distributions (as measured with SEM in stock suspensions) with an average size of 31.6–35.0 nm, except for the 50 nm (+) PS-NPs, which had a larger average size of 50.6 nm (Table 1). The zeta potential measurements in stock suspensions of the 50 nm (+) and 50 nm (−) PS-NPs in water confirmed their positive and negative charges (Table 1). The two types of negatively charged 50 nm PS-NPs (−M and –P) had the same zeta potential (i.e. −27.7 and −27.8 mV), while the zeta potential of the positively charged PS-NPs was 26.6 mV. The neutral PS-NPs had a negative charge of −26.0 mV in water. The size of PS-NPs re-suspended in deionised water, as administered to animals, was measured with DLS. The PS-NPs were monodispersed, and their hydrodynamic diameters ranged from 50.0 ± 0 to 54.3 ± 0.1 nm (Table 1).

**Table 1:** Physicochemical characterization of 50 nm PS-NPs

PS-NPs	SEM^a^ (nm)	DLS^b^ (nm)	Zeta potential^c^ (mV)
50 nm (0)	33.4 ± 12.7	50.0 ± 0.0	−26.0 ± 16.2
50 nm (+)	50.6 ± 9.3	50.3 ± 0.4	26.6 ± 13.9
50 nm (−M)	35.0 ± 15.3	52.7 ± 2.4	−27.7 ± 19.3
50 nm (−P)	31.6 ± 13.6	54.3 ± 0.1	−27.8 ± 17.4

(*0*) neutral PS-NPs, (+) positively charged PS-NPs, (−*M*) and (−*P*) negatively charged PS-NPs from Magsphere and Polysciences, respectively. Data in superscripts a and c from Walczak et al. (2014)

^a^Diameters (nm) of PS-NPs in water, as measured with SEM in stock suspensions (*n* = 80–380)

^b^Hydrodynamic diameters (nm) of PS-NPs in water, as determined by DLS at *t* = 0 h, after re-suspending the PS-NPs in deionised water

^c^Zeta potential (mV) of PS-NPs in water, as determined by a zeta-sizer in stock suspensions at *t* = 0 h

### Fluorescence measurements of blood and organs

**Fig. 1:**
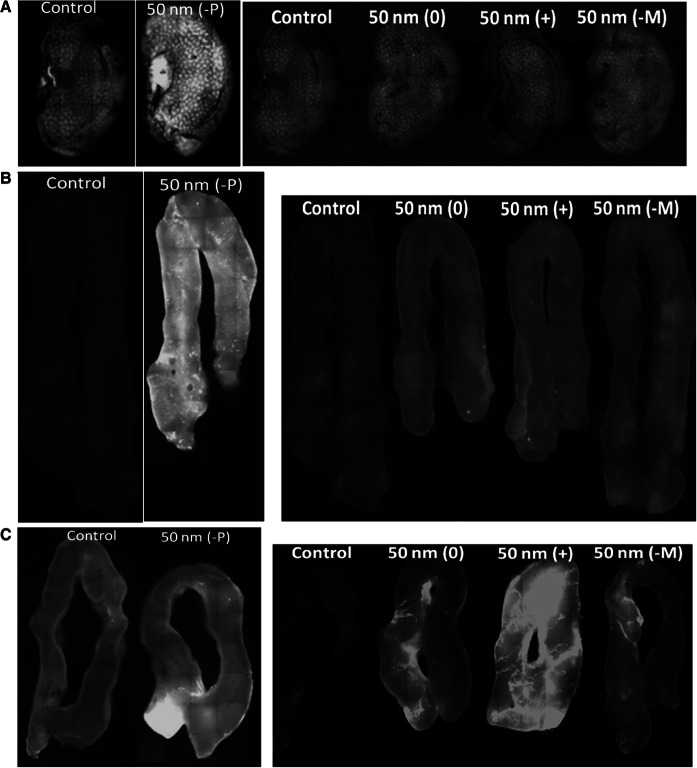
Whole-organ fluorescence following a single oral administration of 125 mg/kg bw PS-NPs. Pictures of kidney (**a**), small- (**b**) and large intestinal walls (**c**) at *t* = 6 h showing fluorescence under the illumination with wavelengths Ex/Em = 470/520 nm or 530/590 nm, for *yellow-green* (−P) PS-NPs and *red* (0, +, −M) PS-NPs, respectively. Control organs were collected from animals treated with only water. (*0*) neutral PS-NPs, (+) positively charged PS-NPs, (−*M*) and (−*P*) negatively charged PS-NPs from Magsphere and Polysciences, respectively. (Color figure online)

**Fig. 2:**
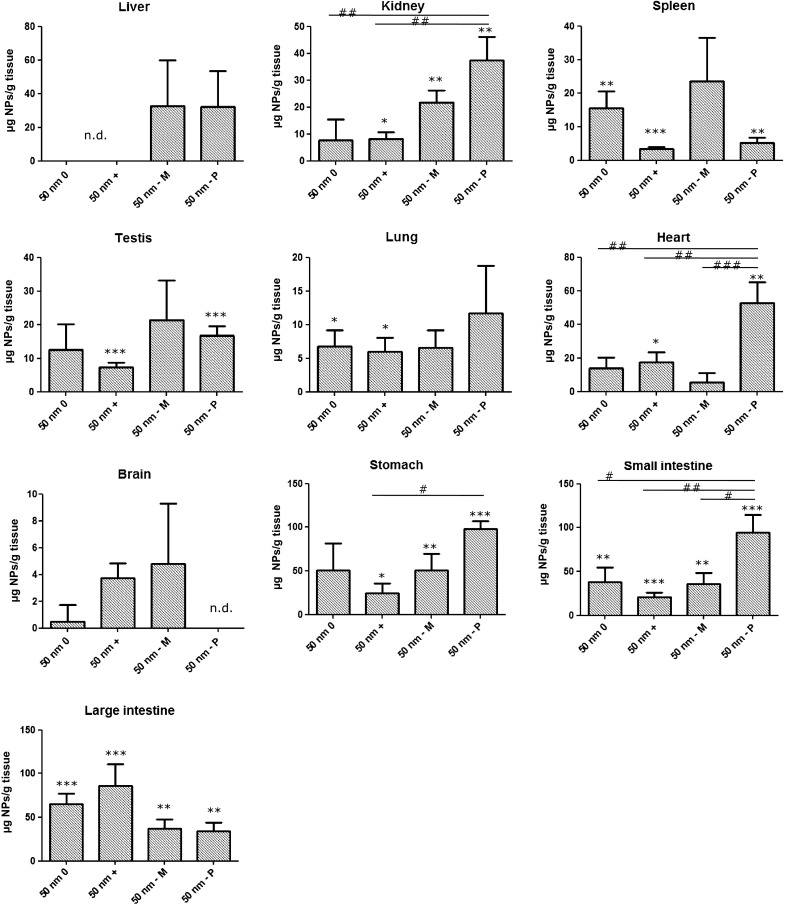
Organ distribution of 50 nm PS-NPs after 6 h from a single oral exposure (125 mg/kg bw), expressed as µg PS-NPs/g tissue, detected in organs from exposed animals. *n.d.* not detectable, (*0*), (−*M*) and (−*P*) negatively charged PS-NPs from Magsphere and Polysciences, respectively. *Error bars* show the standard error of mean (*n* = 5). Significant difference between the blank and exposed organs is illustrated as *p < 0.1; **p < 0.05; ***p < 0.01. Significant difference between different types of PS-NPs is illustrated as ^#^ *p* < 0.1; ^##^ *p* < 0.05; ^####^ *p* < 0.01)

Fluorescence of the collected blood and organs was determined. The concentration of the 50 nm (−P) PS-NPs was high enough for detection in the kidney and small- and large intestinal walls at the appropriate wavelength using fluorescent microscopy, and the concentrations of the 50 nm (0), (+) and (−M) PS-NPs were high enough for detection in the small- and large intestinal walls only (Fig. 1a–c). The fluorescence intensity could not be quantified reliably using whole organs. Therefore, fluorescence intensity was quantified using enzymatically digested organ homogenates, and PS-NP organ concentrations were determined based on standard calibration curves made in each organ. The PS-NP concentrations in the different organs are shown in Fig. 2. Each of the four types of PS-NPs induced a significant increase of fluorescence in at least one of the tested organs, indicating the passage of these PS-NPs through the intestinal wall. In animals exposed to 50 nm (−P) PS-NPs, the concentration of these PS-NPs was significantly increased in kidney (*p* < 0.05), spleen (*p* < 0.05), testis (*p* < 0.01), heart (*p* < 0.05), stomach wall (*p* < 0.000), small intestinal wall (*p* < 0.01) and large intestinal wall (*p* < 0.05). In animals exposed to 50 nm (+) PS-NPs, the concentration of these PS-NPs was significantly increased in kidney (*p* < 0.1), spleen (*p* < 0.01), testis (*p* < 0.01), lung (*p* < 0.1), heart (*p* < 0.1), stomach wall (*p* < 0.1), small intestinal wall (*p* < 0.01) and large intestinal wall (*p* < 0.01). The concentrations of 50 nm (0) and (−M) PS-NPs in the organs were considerably lower than those of 50 nm (−P) and (+) PS-NPs, and they reached significance only in few organs. In the animals exposed to 50 nm (0) PS-NPs, the concentration of these PS-NPs was significantly increased in spleen (*p* < 0.05), lung (*p* < 0.1), small intestinal wall (*p* < 0.05) and large intestinal wall (*p* < 0.01). The concentration of 50 nm (−M) PS-NPs was significantly increased in kidney (*p* < 0.05), stomach wall (*p* < 0.05), small intestinal wall (*p* < 0.05) and large intestinal wall (*p* < 0.05). No PS-NPs were detected in blood samples from any time point.

In some organs, the PS-NP concentration was significantly different between the different types of PS-NPs, depending on their surface modifications. In kidney, the concentration of 50 nm (−P) PS-NPs was significantly higher than that of the 50 nm (+) (*p* < 0.05) and 50 nm (0) PS-NPs (*p* < 0.05). Also in heart, the concentration of 50 nm (−P) PS-NPs was significantly higher than that of 50 nm (+) (*p* < 0.05), 50 nm (0) (*p* < 0.05) and 50 nm (−M) PS-NPs (*p* < 0.01). In the stomach wall, the concentration of 50 nm (−P) PS-NPs was significantly higher than that of 50 nm (+) (*p* < 0.1), and in the small intestinal wall the concentration of 50 nm (−P) PS-NPs was significantly higher than that of 50 nm (+) (*p* < 0.05), 50 nm (0) (*p* < 0.1) and 50 nm (−M) PS-NPs (*p* < 0.1).

**Fig. 3:**
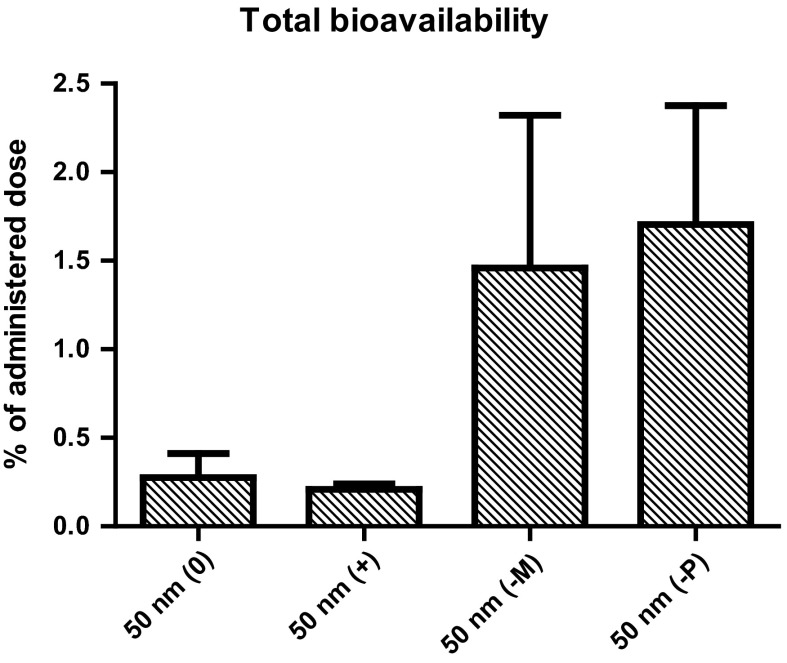
Estimated bioavailability of 50 nm PS-NPs, expressed as a percentage of the administered dose (125 mg/kg bw), calculated by summing up the amounts of PS-NPs detected in all analysed organs, except the stomach- and intestinal walls and brain. (*0*) neutral PS-NPs, (+) positively charged PS-NPs, (−*M*) and (−*P*) negatively charged PS-NPs from Magsphere and Polysciences, respectively. *Error bars* show the standard error of mean (*n* = 5)

The overall bioavailability of PS-NPs was estimated by summing up the amounts of PS-NPs in all measured organs, except the stomach wall and intestinal walls, as PS-NPs present in these organs were most likely the result of direct absorption rather than from uptake from the blood, and except the brain, due to the selectivity of the blood–brain barrier. As shown in Fig. 3, the resulting amount of PS-NPs as a percentage of the administered dose was as low as 0.3 and 0.2 % for 50 nm (0) and (+) PS-NPs, respectively, while the (−M) and (−P) PS-NPs reached bioavailable levels of 1.5 and 1.7 %, respectively. Due to the large variability in the (−M) and (−P) groups, the higher estimated bioavailabilities of these PS-NPs are not significantly different (*p* = 0.2) from the estimated bioavailabilities of the (0) and (+) PS-NPs.

Microscopic observations of tissue slides did not reveal any histopathological changes.

## Discussion

In the present study, we evaluated the bioavailability and biodistribution of differently charged PS-NPs in rats after a single oral administration. Our results show the bioavailability and biodistribution of PS-NPs from the gastrointestinal tract to different organs within 6 h. The highest amounts of PS-NPs were measured in the stomach- and intestinal walls. PS-NPs were detected also in lung, testis, spleen, kidney and heart, meaning that the PS-NPs were systemically available. However, the overall estimated bioavailability was low, ranging from 0.2 to 1.7 % of the administered dose. All types of PS-NPs used in our study had the same size, yet the organ uptake and distribution pattern was different. This shows that the surface charge and chemistry of PS-NPs affected their bioavailability to the organs, as reported before for PS-NPs (Hillery and Florence 1996; Hussain et al. 1997; Jani et al. 1989) and other types of NPs (El-Shabouri 2002; Xiao et al. 2011).

Irrespective of surface charge, all types of PS-NPs were measured in significant amounts in the small- and large intestinal walls, as shown by fluorescence measurements and organ imaging. The intestinal wall has been shown before to be the main site of biodistribution for PS-NPs after oral administration (Hussain et al. 1997; Jani et al. 1992). High levels of PS-NPs measured in intestinal walls in our study could be related with the uptake of PS-NPs in lymphoid tissue associated with these organs, as shown before (Florence et al. 1995; Hillery et al. 1994; Seifert et al. 1996). Also testis, spleen, kidney and heart had considerably high concentrations of PS-NPs, irrespective of the type of administered PS-NPs. These organs, and additionally also liver, have been shown before to be the main organs where PS-NPs (Hussain et al. 1997; Jani et al. 1990) and other types of NPs (Baek et al. 2012; Cho et al. 2013; van der Zande et al. 2012) were distributed after being taken up into systemic circulation after an oral exposure. Strikingly, the liver showed no significant increase in fluorescence above background levels, for any of the used NP types. After sample treatment, all samples still were slightly turbid, which could have distorted the fluorescent signal from these samples potentially resulting in an increased detection limit (Lakowicz 2007). Alternative explanations for the lack of detectable amounts of NPs could be related to the size of the PS-NP. It has been shown that, while liposome NPs smaller than 70 nm accumulated in liver, liposome NPs larger than 200 nm accumulated in spleen (Liu et al. 1992). The PS-NPs used in the present study could possibly agglomerate during the passage through the gastrointestinal tract and as a result become larger than 70 nm. Also another study performed with 50 nm PS-NPs has shown the absence of the NPs in liver after 6 h from a single oral exposure (Jani et al. 1992). Neither PS-NPs were detected in blood at any time point, even with the intervals of half an hour, most likely due to a rapid transport of PS-NPs from the blood circulation to the organs, as shown before (Fabian et al. 2008; Lankveld et al. 2010; Geraets et al. 2014).

Several in vivo oral studies have been performed before with different types of PS-NPs. Table 2 summarizes oral studies performed in rats with different sized and coated PS-NPs. The results from these studies highlight the dependence of uptake and accumulation of PS-NPs on several factors, including the size, surface charge and type of coating material (Araujo et al. 1999; Hillery et al. 1994; Hillyer and Albrecht 2001; Hussain and Florence 1998; Hussain et al. 1997; Jani et al. 1989). In general, smaller PS-NPs were taken up to a higher extent than the larger ones (Jani et al. 1990), the non-ionized more than the carboxylated ones (Jani et al. 1989) and 407 poloxamer-coated more than 188 poloxamer-coated across the GI tract (Hillery and Florence 1996; Hussain et al. 1997).

**Table 2:** Overview of oral studies performed with PS-NPs in rats

NP type	Detection method	Experimental conditions	Dose	Size	Uptake (% of the administered dose)	Ref.
Carboxylated polystyrene nanospheres linked with rhodamine	Fluorescence microscopy observations	Female Sprague–Dawley rats; gavage	1.25 mg/kg bw, daily for 10 days	100 nm, 1 µm	Uptake only semiquantitatively quantified: very low uptake in the stomach wall, small intestinal wall and mesentery node; low uptake in the Peyer’s patch, colon and liver; no NPs in kidney, lungs, heart and spleen	Jani et al. (1989)
Non-ionized polystyrene microspheres linked with fluorescein				100 nm, 500 nm, 1 µm, 3 µm	Low uptake in the spleen, stomach wall and small intestinal wall; moderate uptake in liver and colon; high uptake in the Peyer’s patch and mesentery node; no NPs in kidney, lungs and heart	
Non-ionized polystyrene microspheres linked with fluorescein	Presence of polystyrene was analysed by gel permeation chromatography; measurement of radioactivity of tissues	Female Sprague–Dawley rats; gavage	1.25 mg/kg bw, daily for 10 days	50 nm	Total uptake: 33.7 % Without stomach-, small- and large intestinal walls: 6.6 %^a^ Liver: 3.3 % Spleen: 0.9 % Kidney: 0.2 % Stomach wall: 1.1 % Small intestinal wall: 12 % Large intestinal wall: 14 % No NPs in lungs and heart	Jani et al. (1990)
				100 nm	Total uptake: 26 % Without stomach-, small- and large intestinal walls: 5.9 %^a^ Liver: 3.8 % Spleen: 0.7 % Stomach wall: 0.7 % Small intestinal wall: 3.4 % Large intestinal wall: 16 % No NPs in kidney, lungs and heart	
				300 nm	Total uptake: 9.5 % Without stomach-, small- and large intestinal walls: 2.7 %^a^ Liver: 1.4 % Spleen: 0.2 % Stomach wall: 0.5 % Small intestinal wall: 2 % Large intestinal wall: 4.3 % No NPs in kidney, lungs and heart	
				500 nm	Total uptake: 13.7 % Without stomach-, small- and large intestinal walls: 1.9 %^a^	
				1 µm	Total uptake: 4.6 % Without stomach-, small- and large intestinal walls: 0.8 %^a^	
Non-ionized polystyrene microspheres linked with fluorescein	Fluorescence microscopy observations	Female Sprague–Dawley rats; gavage	12.5 mg/kg, 6 h	50 nm	Uptake only semiquantitatively quantified: Significant uptake in the Peyer’s patches and mesentery nodes; no NPs in liver and spleen	Jani et al. (1992)
				500 nm	Low uptake in the Peyer’s patches; evident uptake in mesentery nodes; no NPs in liver and spleen	
				1 µm	Low uptake in the Peyer’s patches; no NPs in mesentery nodes, liver and spleen	
Carboxylated polystyrene NPs coupled with lectin	Fluorescence microscopy observations; gel permeation chromatography	Female Wistar rats; gavage	12.5 mg/kg, daily for 5 days	500 nm	Total estimated uptake: 37.6 %^a^ Without stomach-, small- and large intestinal walls: 23 % Liver: 2.6 % Spleen: 1.2 % Heart: 0.3 % Kidney: 0.7 % Intestinal wall: 12.8 %	Hussain et al. (1997)
With *N*-acetylchitotetraose					Spleen: 0.42 %	
Non-ionized polystyrene NPs with covalently linked fluorescein, coated with 407 poloxamer	Fluorescence microscopy observations; gel permeation chromatography	Female Sprague–Dawley rats; gavage	14 mg/kg, daily for 5 days	60 nm	Uptake across the GI tract: 3 %: Lymphoid large intestine: 2.0 % Non-lymphoid large intestine: 1 %	Hillery and Florence (1996)
Coated with 188 poloxamer					Uptake across the GI tract: 1.5 %: Lymphoid large intestine: 1.5 %	
Non-ionized polystyrene NPs with covalently linked fluorescein	Fluorescence microscopy observations; gel permeation chromatography	Female Sprague–Dawley rats, 9 weeks, oral gavage	14 mg/kg, daily for 5 days	60 nm	Uptake across the GI tract: 10 %: Lymphoid small intestine: 3.4 % Non-lymphoid small intestine: 2.3 % Lymphoid large intestine: 3.0 % Non-lymphoid large intestine: 2.2 %	Hillery et al. (1994)
Polystyrene NPs, FITC-labelled	Fluorescence microscopy observations	Male Wistar rats: Young (5 weeks); intraduodenally administered, single dose	3.7 × 10^9^ in 1 ml, 6 h	1 µm	Measured in lymph fluid: −2 × 10^−6^ %^a^	Seifert et al. (1996)
		Middle age (5 months)			−2 × 10^−5^ %^a^	
		Old (9 months)			−1.4 × 10^−5^ %^a^	

^a^Calculated from the numbers given in the manuscript

The estimated oral bioavailability that we report here (i.e. 0.2–1.7 %) is lower than that in a previous oral study using 50 nm PS-NPs, where 6.6 % estimated total uptake was reported (Jani et al. 1990) (Table 2). Also the amounts of PS-NPs associated with intestinal tissues that we detected (ranging between 0.38 and 0.74 % depending on the type of PS-NPs, calculated as the sum of the small- and large intestinal walls, data not shown) were lower than the ones reported by others for 60 nm PS-NPs, which varied between 1.5 and 10 %, depending on the type of PS-NPs used (Hillery and Florence 1996; Hillery et al. 1994). The difference between data from the present study and those of other in vivo studies might be due to the use of different exposure conditions, as we exposed the rats for 6 h, while in the previous studies the rats were exposed for 5 or 10 days. Furthermore, the bioavailability values given here were estimated from the amounts of PS-NPs that were measured in a selection of organs and therefore can be underestimated. The differences in the described amounts of NPs that pass the intestinal walls could be further caused by differences in tissue sampling methods and methods of quantifying the concentration of PS-NPs in tissues, and by large interindividual differences as shown before after intraduodenal administration of PS-NPs, where the numbers of particles subsequently found in lymph ducts varied considerably between the different animals (Seifert et al. 1996). However, the amount of 50 nm (−P) PS-NPs that was detected in kidney (0.3 %) was similar to the 0.2 % reported by others for 50 nm PS-NPs (Jani et al. 1990). Comparison of our results from 50 nm (−P) PS-NP to 300 nm PS-NPs in another study shows that the amount of the 50 nm (−P) PS-NPs that we detected in the liver (1.3 %), spleen (0.07 %) and stomach wall (0.54 %) was similar to the 1.4, 0.2 and 0.5 % reported for the liver, spleen and stomach wall of 300 nm PS-NP-treated animals, respectively (Jani et al. 1990). Furthermore, the amount of PS-NPs we detected in the heart (0.17 %) was largely similar to the 0.3 % detected for 500 nm PS-NPs (Hussain et al. 1997). However, our bioavailability values are lower than the extrapolated 23 % that was reported for much larger 500 nm PS-NPs (Hussain et al. 1997). Even larger PS-NPs of 1 µm had a lower uptake than what we report here [2 × 10^−6^ % of 1 µm PS-NPs detected in lymph fluid (Seifert et al. 1996)].

Comparison of the bioavailability values ranging between 0.2 and 1.7 % that we report here, with the translocation values of the same 50 nm PS-NPs in our integrated in vitro digestion and in vitro intestinal model, which ranged from 1.6 to 12.3 % (Walczak et al. 2015), shows lower uptake values in the in vivo model (Table 3). However, a direct comparison should only be performed with caution as the used exposure times in the in vitro and in vivo exposures were different (24 h in vitro and for the in vivo study samples were collected 6 h after administration). In addition, the administered doses used were different. Future kinetic modelling could aid in comparing the results from these experiments. Also the relative order of translocation in vitro (Walczak et al. 2014, 2015) differed from the order of uptake of PS-NPs in vivo. However, the 50 nm (−P) PS-NPs, which translocated to the largest extent in vitro, were also taken up to the largest extent in the present in vivo study, as shown in organs where the PS-NPs concentrations were the highest (i.e. in kidney and heart).

**Table 3:** Comparison of results from in vitro and in vivo experiments measuring intestinal translocation in Caco-2/HT29-MTX cells and systemic uptake in rats, respectively

PS-NPs	In vitro translocation (% of administered dose)^a^	In vivo estimated bioavailability (% of administered dose)
50 nm (0)	9.1 ± 0.8	0.3 ± 0.1
50 nm (+)	4.8 ± 0.7	0.2 ± 0.0
50 nm (−M)	1.6 ± 0.2	1.5 ± 0.9
50 nm (−P)	12.3 ± 1.1	1.7 ± 0.7

(*0*) neutral PS-NPs, (+) positively charged PS-NPs, (−*M*) and (−*P*) negatively charged PS-NPs from Magsphere and Polysciences, respectively

^a^Data from Walczak et al. (2015)

## Conclusion

Our results show that the predicted uptake of PS-NPs from our integrated in vitro model appears to overestimate the actual uptake occurring in the rat in vivo. Therefore, the in vitro model cannot be used for a direct prediction of bioavailability of orally administered PS-NPs in a rat model. However, our model can be used for screening and prioritizing NPs before further in vivo testing for risk assessment and for drug delivery efficacy. Similar to in vitro results, the surface charge and surface chemistry affected the uptake and biodistribution of 50 nm PS-NPs after oral exposure in rats. The negatively charged PS-NPs were present in almost all organs to a much higher extent than the neutral and positively charged PS-NPs, which is in line with the in vitro translocation data of these PS-NPs.

## References

[CR1] Araujo L, Lobenberg R, Kreuter J (1999). Influence of the surfactant concentration on the body distribution of nanoparticles. J Drug Target.

[CR2] Baek M (2012). Pharmacokinetics, tissue distribution, and excretion of zinc oxide nanoparticles. Int J Nanomed.

[CR3] Bhattacharjee S, Marcelis ATM, Zuilhof H, Woutersen RA, Rietjens IMCM, Alink GM (2013). Role of surface charge in bioavailability and biodistribution of tri-block copolymer nanoparticles in rats after oral exposure. Toxicol Res.

[CR4] Bouwmeester H, Brandhoff P, Marvin HJP, Weigel S, Peters RJB (2014). State of the safety assessment and current use of nanomaterials in food and food production. Trends Food Sci Technol.

[CR5] Chaudhry Q (2008). Applications and implications of nanotechnologies for the food sector. Food Addit Contam Part A Chem Anal Control Expo Risk Assess.

[CR6] Cho WS, Kang BC, Lee JK, Jeong J, Che JH, Seok SH (2013). Comparative absorption, distribution, and excretion of titanium dioxide and zinc oxide nanoparticles after repeated oral administration. Part Fibre Toxicol.

[CR8] des Rieux A, Ragnarsson EGE, Gullberg E, Preat V, Schneider YJ, Artursson P (2005). Transport of nanoparticles across an in vitro model of the human intestinal follicle associated epithelium. Eur J Pharm Sci.

[CR9] Durmus-Altun G, Vatansever U, Arzu Vardar S, Altaner S, Dirlik B (2011). Scintigraphic evaluation of small intestinal transit in the streptozotocin induced diabetic rats. Hippokratia.

[CR10] El-Shabouri MH (2002). Positively charged nanoparticles for improving the oral bioavailability of cyclosporin-A. Int J Pharm.

[CR11] Fabian E, Landsiedel R, Ma-Hock L, Wiench K, Wohlleben W, van Ravenzwaay B (2008). Tissue distribution and toxicity of intravenously administered titanium dioxide nanoparticles in rats. Arch Toxicol.

[CR12] Fallingborg J, Christensen LA, Ingeman-Nielsen M, Jacobsen BA, Abildgaard K, Rasmussen HH (1989). pH-profile and regional transit times of the normal gut measured by a radiotelemetry device. Aliment Pharmacol Ther.

[CR13] Fazlollahi F (2011). Polystyrene nanoparticle trafficking across MDCK-II. Nanomedicine.

[CR14] Florence AT, Hillery AM, Hussain N, Jani PU (1995). Nanoparticles as carriers for oral peptide absorption—studies on particle uptake and fate. J Control Release.

[CR15] Genschow E (2002). The ECVAM international validation study on in vitro embryotoxicity tests: results of the definitive phase and evaluation of prediction models. European Centre for the Validation of Alternative Methods. Altern Lab Anim (ATLA).

[CR16] Geraets L, Oomen AG, Krystek P, Jacobsen NR, Wallin H, Laurentie M, Verharen HW, Brandon EFA, de Jong WH (2014). Tissue distribution and elimination after oral and intravenous administration of different titanium dioxide nanoparticles in rats. Part Fibre Toxicol.

[CR17] Hartung T, Luechtefeld T, Maertens A, Kleensang A (2013). Integrated testing strategies for safety assessments. Altex.

[CR18] Hillery AM, Florence AT (1996). The effect of adsorbed poloxamer 188 and 407 surfactants on the intestinal uptake of 60-nm polystyrene particles after oral administration in the rat. Int J Pharm.

[CR19] Hillery AM, Jani PU, Florence AT (1994). Comparative, quantitative study of lymphoid and non-lymphoid uptake of 60 nm polystyrene particles. J Drug Target.

[CR20] Hillyer JF, Albrecht RM (2001). Gastrointestinal persorption and tissue distribution of differently sized colloidal gold nanoparticles. J Pharm Sci.

[CR21] Hussain N, Florence AT (1998). Utilizing bacterial mechanisms of epithelial cell entry: invasin-induced oral uptake of latex nanoparticles. Pharm Res.

[CR22] Hussain N, Jani PU, Florence AT (1997). Enhanced oral uptake of tomato lectin-conjugated nanoparticles in the rat. Pharm Res.

[CR23] Hussain N, Jaitley V, Florence AT (2001). Recent advances in the understanding of uptake of microparticulates across the gastrointestinal lymphatics. Adv Drug Deliv Rev.

[CR24] Jani P, Halbert GW, Langridge J, Florence AT (1989). The uptake and translocation of latex nanospheres and microspheres after oral administration to rats. J Pharm Pharmacol.

[CR25] Jani P, Halbert GW, Langridge J, Florence AT (1990). Nanoparticle uptake by the rat gastrointestinal mucosa: quantitation and particle size dependency. J Pharm Pharmacol.

[CR26] Jani P, McCarthy DE, Florence AT (1992). Nanosphere and microsphere uptake via Peyer’s patches: observation of the rate of uptake in the rat after a single oral dose. Int J Pharm.

[CR27] Kandarova H, Letasiova S (2011). Alternative methods in toxicology: pre-validated and validated methods. Interdiscip Toxicol.

[CR28] Kulkarni SA, Feng SS (2013). Effects of particle size and surface modification on cellular uptake and biodistribution of polymeric nanoparticles for drug delivery. Pharm Res.

[CR29] Lakowicz JR (2007) Principles of fluorescence spectroscopy. Springer, New York. e-ISBN-13 978-0-387-46312-4

[CR30] Lankveld DP, Oomen AG, Krystek P, Neigh A, Troost-de Jong A, Noorlander CW, Van Eijkeren JC, Geertsma RE, De Jong WH (2010). The kinetics of the tissue distribution of silver nanoparticles of different sizes. Biomaterials.

[CR31] Lee CM, Jeong HJ, Yun KN, Kim DW, Sohn MH, Lee JK, Jeong J, Lim ST (2012). Optical imaging to trace near infrared fluorescent zinc oxide nanoparticles following oral exposure. Int J Nanomed.

[CR32] Liu D, Mori A, Huang L (1992). Role of liposome size and RES blockade in controlling biodistribution and tumor uptake of GM1-containing liposomes. Biochim Biophys Acta.

[CR33] Loeschner K, Navratilova J, Kobler C, Molhave K, Wagner S, von der Kammer F, Larsen EH (2013). Detection and characterization of silver nanoparticles in chicken meat by asymmetric flow field flow fractionation with detection by conventional or single particle ICP-MS. Anal Bioanal Chem.

[CR35] Mahler GJ, Esch MB, Tako E, Southard TL, Archer SD, Glahn RP, Shuler ML (2012). Oral exposure to polystyrene nanoparticles affects iron absorption. Nat Nanotechnol.

[CR36] Martinez-Argudo I, Sands C, Jepson MA (2007). Translocation of enteropathogenic Escherichia coli across an in vitro M cell model is regulated by its type III secretion system. Cell Microbiol.

[CR37] PEN (2013) The project on emerging nanotechnologies (28 October 2013 edn). http://www.nanotechproject.org/news/archive/9242/

[CR38] Seifert J, Haraszti B, Sass W (1996). The influence of age and particle number on absorption of polystyrene particles from the rat gut. J Anat.

[CR39] Szentkuti L (1997). Light microscopical observations on luminally administered dyes, dextrans, nanospheres and microspheres in the pre-epithelial mucus gel layer of the rat distal colon. J Control Release.

[CR40] van der Zande M (2012). Distribution, elimination, and toxicity of silver nanoparticles and silver ions in rats after 28-day oral exposure. ACS Nano.

[CR41] Walczak AP (2012). Behaviour of silver nanoparticles and silver ions in an in vitro human gastrointestinal digestion model. Nanotoxicology.

[CR42] Walczak AP, Kramer E, Hendriksen PJ, Tromp P, Helsper JP, van der Zande M, Rietjens IM, Bouwmeester H (2014) Translocation of differently sized and charged polystyrene nanoparticles in in vitro intestinal cell models of increasing complexity. Nanotoxicology: 1–9 10.3109/17435390.2014.944599 25093449

[CR43] Walczak AP, Kramer E, Hendriksen PJ, Helsdingen R, van der Zande M, Rietjens IMCM, Bouwmeester H (2015) In vitro gastrointestinal digestion increases the translocation of polystyrene nanoparticles in an in vitro intestinal co-culture model. Nanotoxicology (Early Online): 1–9. doi:10.3109/17435390.2014.988664 10.3109/17435390.2014.988664 25672814

[CR44] Worth AP, Balls M (2004). The principles of validation and the ECVAM validation process. Altern Lab Anim (ATLA).

[CR45] Xiao K (2011). The effect of surface charge on in vivo biodistribution of PEG-oligocholic acid based micellar nanoparticles. Biomaterials.

